# Reinforcement Learning Assisted Oxygen Therapy for COVID-19 Patients Under Intensive Care

**Published:** 2021-05-19

**Authors:** Hua Zheng, Jiahao Zhu, Wei Xie, Judy Zhong

**Affiliations:** 1. Department of Mechanical and Industrial Engineering, Northeastern University, 360 Huntington Avenue, Boston, MA 02115; 2. Division of Biostatistics, Department of Population Health, New York University School of Medicine, 180 Madison Avenue, New York, NY 10016

**Keywords:** COVID-19, intensive care, reinforcement learning, respiratory failure, oxygen flow rate control

## Abstract

**PURPOSE:**

Patients with severe Coronavirus disease 19 (COVID-19) typically require supplemental oxygen as an essential treatment. We developed a machine learning algorithm, based on a deep Reinforcement Learning (RL), for continuous management of oxygen flow rate for critical ill patients under intensive care, which can identify the optimal personalized oxygen flow rate with strong potentials to reduce mortality rate relative to the current clinical practice.

**METHODS:**

We modeled the oxygen flow trajectory of COVID-19 patients and their health outcomes as a Markov decision process. Based on individual patient characteristics and health status, a reinforcement learning based oxygen control policy is learned and real-time recommends the oxygen flow rate to reduce the mortality rate. We assessed the performance of proposed methods through cross validation by using a retrospective cohort of 1,372 critically ill patients with COVID-19 from New York University Langone Health ambulatory care with electronic health records from April 2020 to January 2021.

**RESULTS:**

The mean mortality rate under the RL algorithm is lower than standard of care by 2.57% (95% CI: 2.08–3.06) reduction (P<0.001) from 7.94% under the standard of care to 5.37 % under our algorithm and the averaged recommended oxygen flow rate is 1.28 L/min (95% CI: 1.14–1.42) lower than the rate actually delivered to patients. Thus, the RL algorithm could potentially lead to better intensive care treatment that can reduce mortality rate, while saving the oxygen scarce resources. It can reduce the oxygen shortage issue and improve public health during the COVID-19 pandemic.

**CONCLUSION:**

A personalized reinforcement learning oxygen flow control algorithm for COVID-19 patients under intensive care showed substantial reduction in 7-day mortality rate as compared to standard of care. In the overall cross validation cohort independent of the training data, mortality was lowest in patients for whom intensivists’ actual flow rate matched the RL decisions.

## Introduction

Over the course of the past year, the rapid global spread of severe acute respiratory syndrome coronavirus-2 (SARS-CoV-2), has motivated multidisciplinary investigation efforts to identify effective medical management against coronavirus disease 2019 (COVID-19). Respiratory distress, including mild or moderate respiratory distress, acute respiratory distress syndrome (ARDS) and hypoxia, is a common complication of COVID-19 patients and the therapy of COVID-19 is guided by the knowledge and experience of moderate-to-severe ARDS treatment [1]. Oxygen therapy is recommended as the first-line therapy of COVID-19-induced respiratory and hypoxia by the Centers for Disease Control and Prevention (CDC) and the World Health Organization (WHO). Oxygen therapy consists of different kinds of supplemental oxygen therapies including nasal cannula, simple mask, venturi mask, non-rebreather masks and high flow oxygen systems. The key factor in different supplemental oxygen methods is the setting of different levels of oxygen flow rates [2]. Thus, the selection of appropriate oxygen flow rate is a crucial decision in COVID-19 treatment. To improve the treatment efficiency, the administration of oxygen therapy should be determined by the severity of COVID-19-induced respiratory failure, incorporating the uncertainties in measurements of patient health status and prediction of individual’s outcomes to the oxygen decisions. It certainly requires a comprehensive investigation of the optimal and personalized oxygen flow rate. Our research aims to explore the effective oxygen therapy for COVID-19 patients based on the continuous respiratory support and vital signs monitoring.

Remarkable advances in oxygen therapy have been made in a short period in the treatment of COVID-19 pneumonia [3, 4]. However, respiratory failure still remains the leading cause of death (69.5%) for SARS-CoV-2 [5]. Thus, we provide an artificial intelligence (AI) oxygen flow control algorithm, based on deep reinforcement learning (RL), which is able to suggest personalized optimal oxygen flow rate for COVID-19 patients based on the knowledge of patient health status estimated from patients’ electronic health records (EHRs). Reinforcement learning has been successfully applied in the past to different healthcare problems such as multimorbidity management [6], HIV therapy [7], cancer treatment [8], and anemia treatment in hemodialysis patients [9]. For critical care, given the large amount and granular nature of electronic recorded data, RL is well suited for providing sequential optimal treatment recommendation and improving outcomes for new ICU patients [10]. Recent studies include treatment strategies for sepsis in intensive care [11] and personalized regime of sedation dosage and ventilator support for patients in Intensive Care Units (ICUs) [12].

Focusing on RL based oxygen flow rate control (RL-oxygen), we studied its impact on mortality in COVID-19 patients with respiratory failure. The evolution of patients’ ICU histories, including treatment, vitals and health outcomes, was modeled using a Markov decision process (MDP) [11, 13]. At each decision epoch, based on the state (observed patient characteristics, including age, sex, race, smoking status, BMI and comorbidity diagnoses, 36 daily observed lab test values and 6 unique vitals), RL selected an oxygen flow rate (ranged from 0 to 60 L/min), and obtained a reward defined based on patient’s 7-day survival. Then, following the oxygen flow rate suggested by RL policy, an estimated mortality rate was predicted to compare with the mortality rate in actual practice.

## Methods

### Study Design and Participants

Our research team used a retrospective cohort of the New York University Langone Health (NYULH) EHR data on COVID-19 patients to derive and validate the RL algorithm. Eligible patients have had positive COVID-19 PCR test and had oxygen therapy in hospital between March 1st 2020 to January 19th 2021. We excluded COVID-19 patients aged below 50 and not been hospitalized as these lacked consistent documentation of vital signs, treatment and laboratory tests. This study was approved by the NYULH IRB and the data were de-identified to ensure anonymity.

For each patient, we had access to demographic data, including age, sex, race, ethnicity and smoking status, ICU admit and discharge information, in hospital living status, comorbidities, treatment and laboratory test data. The comorbidities, including hyperlipidemia, coronary artery disease, heart failure, hypertension, diabetes, asthma or chronic obstructive pulmonary, dementia and stroke, are defined based on International Classification of Diseases (ICD)-10 diagnosis codes. To reduce the feature dimensionality, we selected 36 laboratory tests based on two criteria: (1) less than 28% missing values and (2) COVID-19 related. In specific, we explore the associations between laboratory tests and COVID-19 based on existing literature and clinical findings. For example, recent study has shown that a reduced estimated glomerular filtration rate (eGFR), low platelet count, low serum calcium level, increased white blood cell count, Neutrophil-to-lymphocyte ratio (NLR), and red blood cell distribution width-coefficient of variation (RDW-CV) are related to high risk of severity and mortality in patients with COVID-19 [14–18]. Additionally, some research suggests well-controlled blood glucose is associated with the lower mortality in COVID-19 patients with Type-2 diabetes [19] and continuous renal potassium level has correlation of hypokalemia, which is common among patients with COVID-19 [20]. Arterial blood gas analysis, including pH, Oxyhemoglobin saturation (*SaO*_2_), oxygen saturation (*SpO*_2_), partial pressure of oxygen (*PaO*_2_) and bicarbonate (*HCO*_3_), is commonly used biomarkers measuring the severity of ARDS [21, 22].

In this study, we employed leave-one-hospital-out validation to evaluate the model performance. The whole dataset was divided into 4 batches by the hospital and then we take one batch as validation set and the rest as training set in each simulation.

### RL algorithm Overview

We model patient health trajectory and the clinical decisions during a course of intensive care over a period of ICU stay by a Markov decision process (MDP) with state, action and reward. The state of a patient includes the observed patient demographics, vital signs and laboratory test at each time. The action refers to oxygen flow rate. As a consequence of a sequence of actions, the patient receives a reward if he/she survives in the next 7 days, otherwise a penalty to death will be given. The cumulative return is defined as the discounted sum of all rewards of each patient received during the ICU stay. RL is designed to maximize the cumulative return by making optimal actions at each time through an off-policy algorithm, named Deep Deterministic Policy Gradient (DDPG) [23]. Briefly speaking, DDPG learns a scoring rule which evaluates the recommended oxygen flow rate given a patient’s health state and then uses such a rule to improve the decision making by optimizing the score. The intrinsic design of RL provides a powerful tool to handle sparse and time-delayed reward signals, which makes them well-suited to overcome the heterogeneity of patient responses to actions and the delayed indications of the efficacy of treatments [11].

The details of state, action and reward are listed as following:

- State: observed patient’s characteristics at each time with information, including demographics, COVID-19 lab tests and vital signs.
- Action: oxygen flow rate ranged from 0 L/min to 60 L/min.
- Reward: the reward of an action is measured by its associated ultimate health outcome given the patient health state. Similar to [11], we used in-hospital mortality as the system-defined penalty and reward. When a patient survived, a positive reward was released at the end of the patient’s trajectory (i.e., a `reward’ of +15); a negative reward (i.e., a `penalty’ of −15) was issued if the patient died. We find such a reward can propagate the final health outcome backward to each decision over the period so that RL can predict long-term effect and dynamically guide the optimal oxygen flow treatment.
- Discount factor: determines how much the RL agents balance rewards in the distant future relative to those in the immediate future. It can take values between 0 and 1 [13]. After considering the ICU stay tends to be short and also conducting side experiments, we chose a value of 0.99, which means that we put nearly as much importance on late deaths as opposed to early deaths for each recommended oxygen flow rate.

### Model Evaluation

We evaluated the RL-recommended oxygen therapy by comparing its effect with the observed one on the cohort from each validation hospital. At each decision time, the RL algorithm recommends an oxygen flow rate for the patient. If the absolute difference of recommended and the observed oxygen flow rate is less than 10 L/min, we say that RL is “consistent” with the critical care physicians.

When RL is discrepant with the oxygen flow rate used by physicians, the efficacy of the RL-recommended oxygen therapy is not directly observed. The problem then becomes how to assess the health outcomes in the future after taking RL recommendation. For this reason, we predicted the outcome of the RL-recommended treatment using Cox proportional hazards model, a regression model commonly used for investigating the association between the survival probability of patients during a time period and predictor variables of interest in medical observational studies [24, 25]. In short, a patient was labeled as “alive” if he/she survived after a treatment within seven days, otherwise, labeled as “deceased”. Then we fitted a Cox survival model with demographics, vital signs and lab tests as predictors and evaluated the effect of decision using the leave-one-hospital-out validation.

To assess the performance of the survival models, we compared predicted and observed outcomes (7-day living status) using 4 metrics: similarity, accuracy, Chi-squared test, and concordance index. Overall, the cosine similarity between predicted and actual survival is greater than 99.9% and concordance indices are 0.83. Both metrics indicate that the predictive model can effectively estimate unobserved health outcomes. Moreover, the paired Chi-squared test (p-value < 0.0001) shows no significant difference between true and predicted survival.

## Results

Overall, 1,362 patients in NYULH EHR samples had a PCR-based COVID-19 diagnosis between March 2020 to January 2021. The demographic and clinic characteristics summary of the analysis cohort is shown in Table 1. Overall, patients’ mean age is 69.7 and the cohort is comprised of 483 females (35.2%). On average, COVID-19 patients showed BMI of 28.61 kg/m2, pO2 (partial pressure of oxygen) of 104.8 mmHg, SaO2 (Oxygen saturation in arterial blood) of 94.1% and SBP of 123.4 mmHg. Hypertension, hyperlipidemia, diabetes and coronary artery disease are top 4 common comorbidities for COVID-19 patients aged above 50, diagnosed in 85.2%, 71.8%, 51.4% and 41.2% patients respectively. The median hospital stay duration was 2.9 days since COVID-19 diagnosis (interquartile range [IQR] 0.52–12.2 days). We trained the RL algorithms using patients from each 3 hospitals, and then assessed their performance using the remaining hospital encounters.

The performance of the RL-oxygen is summarized in Table 2. Overall, the RL-oxygen algorithm shows superior performance to the clinical practice of oxygen therapy for COVID-19 patients. The overall 7-day estimated mortality under Physician prescribed oxygen was 7.94% (95% CI: 7.41–8.47), while overall estimated mortality under RL-oxygen was 5.37% (95% CI: 4.94–5.80), showing a 2.57% (95% CI: 2.08–3.06) reduction (P<0.001). In addition, Table 2 depicts the characteristics of oxygen flow rate following the recommendations from both RL-oxygen and physicians. On average, the overall oxygen flow rate was 1.28 L/min (95% CI: 1.14–1.42) lower than the rate actually delivered to patients.

The efficacy of the RL prescriptive algorithms was consistently observed across age, gender, BMI, and comorbidity subgroups (Table 2). Demographically speaking, COVID-19 patients of age older than 75 observed higher efficacies from RL-oxygen recommended oxygen therapy than physician’s recommendations as compared to the observed efficacies in patients of age 75 and younger. For example, 7-day estimated mortality rate under RL-oxygen for patients of age older than 80 was 5.87% (95% CI: 4.67–7.07) lower than under physician’s therapy. In contrast, the 7-day estimated mortality rate under RL-oxygen was 0.55% (95% CI: 0.39–0.71) lower than that under physicians’ therapy for patients aged between 50 and 65. Table 2 also shows that the RL-oxygen tends to be more effective for patients with comorbidities. Especially for COVID-19 patients with Asthma or chronic obstructive pulmonary, Dementia and Stroke, RL-oxygen reduced the 7-day mortality by 5.69%, 5.11% and 3.8% respectively on average.

**Fig 1.**
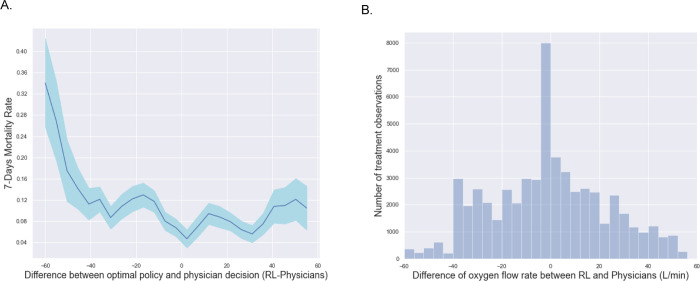
(A) Comparison of the estimated 7-days mortality rates (y-axis) varying with the difference between the oxygen flow rate recommended by the RL optimal policy and that administered by doctors (x-axis) averaged over all time points per patient. The shaded area represents the 95% confidence interval. The smallest oxygen difference is mainly associated with the lowest 7-days mortality rates. The further away the dose received was from the suggested oxygen flow rate, the worse the outcome. (B) The histogram of oxygen flow rate difference between RL-oxygen and physicians (labels on vertical axis).

We further studied 7-days mortality when the actually administered oxygen flow rates differed from the oxygen flow rate suggested by the RL-oxygen in Fig. 1. It shows how the observed mortality changes with the flow rate difference between RL-oxygen and physicians. This phenomenon suggests that increasing differences between the RL-oxygen and the observed delivering oxygen was associated with increasing observed mortality rates in a rate-dependent fashion. When the difference is minimum, we obtain the lowest 7-day mortality rates of 1.7%. Another observation from Fig. 1(A) is that the mortality rate increases when the RL-oxygen flow rate is lower or higher than the one from physicians. It suggests that both the oxygen deficit (lower oxygen flow rate than RL-oxygen recommendation) and the oxygen excess are sup-optimal for patients’ outcomes. We observed a trend that RL-oxygen was in general lower than what prescribed by the physicians and might result in better outcomes under lower flow rate. It suggests that oxygen flow rates prescribed by doctors tend to be excessively high for some patients.

**Fig 2.**
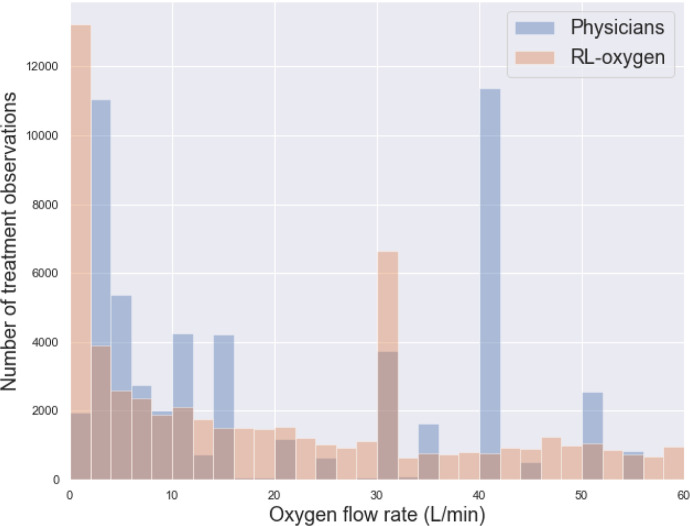
Oxygen delivery by RL versus critical care physicians. Histogram of oxygen flow rate delivered to COVID-19 patients; blue bar indicates physician and orange bar indicates RL-oxygen.

Last, we observed that the RL-oxygen and physicians recommended consistent flow rates in the majority of times; see Fig. 1B. The overall distribution of oxygen flow rates recommended by RL-oxygen and physicians are presented in Fig. 2. It depicts how many measurement times each oxygen flow rate was recommended by RL-oxygen and physicians. In twenty-nine percent of the time, the patients actually received an oxygen flow close to the suggested rate within 5 L/min while forty-four percent of the time, the difference between the administered and suggested oxygen flow rates are within 10L/min. Since the high-flow nasal oxygen (HFNO) therapy often increases flow rate in increments of 10 L/min up to 60 L/min [26], it suggests that RL-oxygen is consistent with physicians about 40–50% of the time.

## Discussion

We used a RL approach to learn an optimal policy to continuously control the oxygen device for critically ill patients with COVID-19 who require the oxygen therapy. As most people who become seriously unwell with COVID-19 have an acute respiratory illness [27, 28], our algorithm has strong potential to improve individual health outcomes and reduce COVID-19 mortality rate caused by respiratory failure. We designed the reward as the ultimate health outcome which is used to assess the performance of oxygen flow decisions along the treatment trajectory. As such, the reinforcement learning approach took uncertain outcomes and long-term treatment effects into consideration and made it smarter in understanding the long impact of an early decision on the final outcomes.

Our analysis suggests the current practice remains some potential to be improved as actual oxygen flow rate administered by intensivists showed more than fifty percent discrepancy with RL-oxygen recommendations. Importantly, we observe that RL-oxygen tends to prescribe lower oxygen flow rate than physician’s prescribed rates, but leads to better outcomes. This finding is especially important in the context of the ongoing and persistent medical oxygen shortages in some developing regions. As COVID-19 patient-care protocols have evolved, medical-grade oxygen is still considered essential to treatments for critically ill patients. In regions such as Africa, the Middle East, and Asia, the surge in demand for medical oxygen to treat COVID-19 exacerbates preexisting gaps in medical-oxygen supplies, leading to substantial supply shortages.

Our analysis also identified some clinical patterns that RL-oxygen particularly works well. For example, patients with high risk (i.e., of age older than 75) observed higher efficacies than patients aged between 50 and 75 by using relatively lower averaged oxygen flow rate than actually administered. RL-oxygen also recommends a higher averaged oxygen flow rate may improve the health outcomes for patients aged from 50 to 65.

Although our evaluation methodology controls for several confounding factors and shows high validation accuracy, sample scarcity and large proportion of missing value may increase estimation uncertainty and affect the treatment recommendations. A larger training data is necessary to cover more of the state space and improve the policy optimization. Moreover, the COVID-19 cohort from NYULH may not be representative of the U.S. COVID-19 population or the oxygen clinical practices in other countries. To ultimately validate the efficacy of the RL algorithms, randomized clinical trials with patients randomly assigned to RL and clinician mechanism would be needed.

## Conclusion

Through analyzing the EHR data from multiple ambulatory care centers, we demonstrated the feasibility of using reinforcement learning based oxygen therapy to improve the intensive care for COVID-19 patients. The RL-oxygen showed medium concordance (44%) with the current practice of critical care physicians. For all COVID-19 patients requiring oxygen therapy, RL recommendations significantly reduce mortality rate compared to the current practice. The algorithm has potential to be integrated into the clinical decision support system and assist physicians to provide the timely personalized recommendations of oxygen flow rate for COVID-19 patients in ICU.

## Supplementary Material

1

## Figures and Tables

**Table 1:** Demographics and clinical characteristics of NYULH-EHR patients with COVID-19.

Demographics and clinic characteristics	Number of Patients (N=1,372)
Age (years, Mean (SD))	69.72 (10.75)
Male (N (%))	64.49 (0.47)
Race (N(%))	
African American	180 (13.12)
Native American	5 (0.36)
Asian	120 (8.75)
Caucasian (White)	730 (53.21)
Multiple Races	19 (1.39)
Other Races	266 (19.39)
Race Unknown or Patient Refused	53 (3.86)
Smoking ((N%))	1,043 (6.88)
Never	735 (53.57)
Former	443 (32.29)
Current	55 (4.01)
Not asked	139 (10.13)
Body Mass Index (kg/m^2^, Mean (SD)	28.61 (6.74)
Hyperlipidemia (N(%))	978 (71.75)
Coronary artery disease (N(%))	562 (41.23)
Heart failure (N(%))	406 (29.79)
Hypertension (N(%))	1161 (85.18)
Diabetes (N(%))	701 (51.43)
Asthma or chronic obstructive pulmonary (N(%))	217 (15.92)
Dementia (N(%))	133 (9.76)
Stroke (N(%))	195 (14.31)

Categorical variables are summarized with frequencies (percentages) unless otherwise indicated. Continuous variables are summarized as the mean (standard deviation) of biomarkers.

**Table 2:** Subgroup comparison of 7-day estimated mortality obtained using RL-oxygen algorithm and critical care physician decision guidance.

Subgroups	Estimated Mortality (%)		Average Oxygen (L/min)	
	RL-oxygen	Physician	RL-oxygen	Physician
Overall	5.37 (0.22)	7.94 (0.27)	19.24 (0.07)*	20.52 (0.07)
Male	6.13(0.12)*	8.53(0.14)	21.20(0.09)*	22.66(0.09)
Female	2.18(0.11)*	2.99(0.12)	6.33(0.07)	6.41(0.07)
Age				
50 to 65	1.19(0.08)*	1.74(0.09)	25.54(0.12)*	22.27(0.12)
65 to 75	4.13(0.14)*	5.43(0.16)	19.63(0.12)*	22.73(0.12)
75 to 80	14.76(0.3)*	20.39(0.34)	19.79(0.14)*	21.45(0.16)
≥80	15.86(0.57)*	21.73(0.65)	14.28(0.18)*	18.96(0.26)
Body Mass Index (kg/m^2^)				
<25	7.74(0.18)*	11.10(0.21)	19.27(0.11)*	20.58(0.12)
25 to 30	7.38(0.19)*	9.21(0.21)	23.39(0.13)*	24.5(0.14)
30–35	2.72(0.15)*	5.30(0.21)	22.91(0.16)*	21.42(0.17)
≥35	5.35(0.28)*	5.44(0.28)	19.53(0.18)*	22.78(0.21)
Hyperlipidemia	7.43(0.13)*	9.47(0.14)	20.11(0.09)*	20.94(0.09)
Coronary artery disease	8.55(0.18)*	11.39(0.21)	18.13(0.12)*	20.04(0.11)
Heart failure	11.25(0.23)*	12.59(0.25)	18.35(0.11)	18.22(0.13)
Hypertension	6.96(0.11)*	8.79(0.13)	21.2(0.08)	21.25(0.08)
Diabetes	7.73(0.15)*	8.25(0.15)	25.22(0.11)*	20.31(0.1)
Asthma or chronic obstructive pulmonary	11.98(0.32)*	17.67(0.38)	15.57(0.15)*	19.68(0.18)
Dementia	10.71(0.46)*	15.82(0.56)	15.57(0.23)*	14.19(0.23)
Stroke	9.15(0.31)*	12.95(0.37)	21.78(0.15)*	15.94(0.19)

Categorical variables are summarized with frequencies (percentages) unless otherwise indicated. Continuous variables are summarized as the mean (standard error) of biomarkers.

* Variables indicate RL-oxygen is significantly different from physicians (p-value<0.001).
