# Supplementary material for: Reinforcement Learning Assisted Oxygen Therapy for COVID-19 Patients Under Intensive Care
Source: ArXiv. 2021 May 19:arXiv:2105.08923v1. Preprint. [Version 1] (PMC8142656)
Supplement: Supplementary file 1 [file NIHPP2105.08923V1-supplement-1.pdf]

Title: Reinforcement Learning Assisted Oxygen Therapy for COVID-19 Patients Under Intensive Care

Supplemental Material

Cox Proportional Hazards Model

Cox proportional hazards model [1] is a regression model commonly applied for investigating the association between the risk factors and survival time of patients. Its primary output is the mortality rate of patients. In this study, researchers used multivariable Cox PH model to predict the mortality rate. The input variables in the Cox PH model include: (1) decision, i.e., the oxygen flow rate of the oxygen therapy; and (2) the risk factors associated to COVID-19, such as age, hypertension and diabetes. Besides, we set up a 7-day time window to estimate the mortality rate and evaluate the efficiency of a given oxygen therapy. Specifically, we formalize the variables in Cox PH model as follows.

- t: denotes the duration since the patient admission time;
- $S(t)$: denotes the survival rate of patients at time t;
- x: denotes the predictor variables related to the survival rate;
- β: denotes the coefficient of the corresponding variables.

The objective of Cox PH model used to predict the survival rate is given by

$$S(t \mid x) = \exp\left(-\int_0^t \lambda(z|x)dz\right), \qquad (1)$$

where the hazard at time t for an individual with covariates x (not including a constant) is assumed to be

$$\lambda(t \mid x) = \lambda_0(t)\exp(x^\top \beta).$$

In this model， $\lambda_0(t)$ is a baseline hazard function that describes the risk for individuals with $x = 0$, and exp $(x^\top \beta)$ is the relative risk, a proportionate increase or reduce in risk, associated with the set of characteristics x . Note that the increase or reduce in risk is the same at all duration t. Given health state x of a patient, we predict the 7-day mortality rate by using $1 - S(7 \mid x)$.

Feature Selection

Feature selection is significant for establishment of Cox PH model since unrelated risk factors and the high multilinearity between predictor variables will cause low concordance and impact on the prediction. In addition to the selected 36 laboratory tests (see **Study Design and Participants**), we also included 25 additional demographic

predictor variables, 2 vital signs (temperature and systolic blood pressure) and oxygen flow rate. Since there were high correlations between the selected features, we conduct feature selection based on Pearson correlation to preclude multilinear features. Basically, we found the high linear correlation (>0.7) existing in each group of features, including: (1) red blood cell distribution width-coefficient of variation (RDW-CV) and red cell volume distribution width-standard deviation (RDW-SD); (2) eGFR and creatinine; (3) red blood cell count, hemoglobin and hematocrit; (4) neutrophils and lymphocytes; and (5) SpO2, oxyhemoglobin and methemoglobin. We selected the first predictor in each group and removed the rest: RDW-CV, eGFR, red blood cell count, neutrophils and SpO2.

For the rest feature selection, we used the elastic net regularization [2] with grid search [3] to select the features. The procedure is shown as follows.

1. We create a grid of possible values for regularizers in cross-product of L1 and L2 penalty values ranging in [0.01, 0.02, 0.04, 0.06, 0.08]. It results in 25 different combinations in total, i.e., $(0.01,0.01), (0.01,0.02) \ldots, (0.08,0.06), (0.08,0.08)$.
2. For each combination of L1 and L2 penalty values, we fitted a Cox model with elastic net regularization and recorded the performance measured by the concordance score.
3. Finally, we chose the best L1 and L2 regularizers with the best performance.

In the study, the selected coefficients of L1 and L2 regularizers are 0.04 and 0.02 respectively.

Training Process

We apply leave-one-hospital-out cross validation to evaluate the models and predict the 7-day survival probability to assess the performance of RL-oxygen models. To train the general model (including data from different hospitals), we randomly select 80% of the cohort as training set and the rest 20% as test set. The coefficient of predictor variables of general Cox PH model shows in table S.1.

Table S.1 Selected features for Cox proportional-hazards model.

Feature name	Coefficient	SE	95% CI	p-value
Age, years	0.02	0.00	0.02	<0.001
Anion gap, mEq/L	0.03	0.02	[0.02, 0.03]	<0.001
Blood urea nitrogen, mg/dL	0.00	0.00	0.00	<0.001
Serum calcium, mg/dL	-0.19	0.01	[-0.22, -0.16]	<0.001
PaCO2, mm Hg	-0.01	0.00	[-0.02, -0.01]	<0.001
Eosinophils, cells/µL	-0.04	0.01	[-0.05, -0.02]	<0.001
HCO3, mEq/L	-0.01	0.00	[-0.02, -0.01]	<0.001
Mean platelet volume, fL	0.05	0.01	[0.03, 0.07]	<0.001
Nucleated red blood count, /100 WBC	0.08	0.02	[0.04,0.12]	<0.001
PH	-1.86	0.11	[-2.08, -1.64]	<0.001
Inorganic phosphorus, mg/dL	0.03	0.01	[0.02, 0.05]	<0.001
PaO2, mmHg	0.00	0.00	0.00	<0.001
Potassium, mEq/L	0.15	0.02	[0.11, 0.18]	<0.001
RDW-CV, %	0.06	0.00	[0.05, 0.06]	<0.001
White blood cell count	0.01	0.00	0.01	<0.001
Oxygen flow rate, L/min	0.01	0.00	0.01	<0.001

The confidence interval is replaced by the coefficient estimates if the SE is smaller than 0.01

Reinforcement Learning Algorithms

A Markov decision process (MDP) was used to model the decision-making process and approximate individual patient health trajectories. We formalize the MDP by the tuple (S, A, P, r, γ), where

- S: denotes a finite set of states, typically including patients' demographic information, ICU admit and discharge information, comorbidities, treatment, laboratory tests and living status;

- A: denotes action space, i.e., oxygen flow rate;
- $P(s'|a, s)$: represents the state transition probability model that taking action a in state s at time t will lead to state s' at time $t + 1$ (i.e., the patient's health state changes to s' at $t + 1$ after taking oxygen therapy with flow rate a at time t), which describes the dynamics of the treatment process;
- r: represents the immediate reward received for transitioning to state s' . Transitions to desirable states yield a positive reward, and reaching undesirable states generates a penalty.
- γ: denotes the discount factor, which makes immediate rewards more valuable than long-term rewards and determines the temporal impact of the current action. The greater γ indicates longer impact of current therapy action.

The process is observed at discrete time steps. In each time t, the agent observes the current state $s_t \in S$. Then, we choose an action $a_t \in A$ (i.e., oxygen flow rate), the patient health conditions moves to a new state s_{t+1}, and we get a

reward signal r_{t+1} associated with the one-step transition (s_t, a_t, s_{t+1}). The oxygen flow rate decision making strategy is called the *policy*, denoted by a mapping π from state space S to action space A, i.e., $a_t = \pi(s_t)$. The performance of a policy is measured using the value function

$$V^{\pi}(s) = \mathrm{E}[\sum_{t=1}^{\infty} \gamma^t r_t | s_0, \pi] \tag{2}$$

which is defined as the expected cumulative discounted reward starting with state s_0, given that policy π is used to make decisions. Then, the goal of a reinforcement learning agent is to learn the optimal policy π^* which maximizes the expected cumulative discounted reward, that is, $V^{\pi}(s)$.

The reward $r_1(s_t, s_{t+1})$ at each time t for each disease is defined as follows,

- If patient stay alive, $r_t = 0$;
- If patient discharged, $r_t = 15$;
- If patient died, $r_t = -15$;

Learning the Optimal Policy

We utilize the Deep Deterministic Policy Gradient (DDPG) to concurrently learn the Q-function and optimal policy. In each iteration, we use off-policy data and the Bellman equation to learn the Q-function, and then learn the optimal policy.

This approach is closely connected to *Q-learning*. In reinforcement learning, many algorithms focus on estimating the so-called "Q-function" $Q^{\pi}(s, a)$ of a policy π. The Q-function represents the expected value of state-action pairs, and it can be connected to the value function through the equation

$$V^{\pi}(s) = \max_a Q^{\pi}(s, a). \tag{3}$$

DDPG interleaves learning an approximator to $Q^{\pi^*}(s, a)$ with learning an approximator to the optimal policy $\pi^*(s)$. For continuous action space, the function $Q^{\pi}(s, a)$ is presumed to be differentiable with respect to the action argument. The Q-function measures the expected return or discounted sum of rewards obtained by following the policy π and taking action $a = \pi(s)$. The *optimal* Q-function is then defined as the maximum return that can be obtained starting from state s, taking action a, and following the optimal policy π^* thereafter. The optimal Q-function is known to obey the following Bellman optimality equation:

$$Q^{\pi^*}(s, a) = \mathrm{E}_{s'}[r(s, a) + \gamma \max_{a'} Q^{\pi^*}(s', a')] \tag{4}$$

where the next state s' is sampled from the state transition distribution, denoted by $P(\cdot\,|s, a)$.

We use a nonlinear function, such as a neural network with parameters θ, to approximate the state-action value function, i.e., $Q^{\pi}(s, a) \approx Q^{\pi}(s, a; \theta)$. Such a neural network is called a Q-network [29]. Let $a(s) = \pi_{\phi}(s)$ denote the deterministic policy function parameterized by ϕ. The Q-function is trained by minimizing the approximation difference (loss function) between the left- and right-hand side in Eq. (4), i.e.,

$$L(s,a) = \frac{1}{2}\mathrm{E}_{s'\sim p(\cdot|s,\,a)}\left[\left(Q^{\pi}(s,a;\theta) - r(\mathrm{s},a) - \gamma \max_{\pi} Q^{\pi}\left(s', \pi_{\tilde{\phi}}(s'); \tilde{\theta}\right)\right)^2\right], \tag{5}$$

or equivalently,

$$L(s,a) = \mathrm{E}_{s'\sim p(\cdot|s,\,a)}\left[\ell_{\theta}(s,a,\pi_{\tilde{\phi}}(s'))\right], \tag{6}$$

and

$$\ell_{\theta}(s,a,s') = \frac{1}{2}\left(Q^{\pi}(s,a;\theta) - r(\mathrm{s},a) - \gamma \max_{\pi} Q^{\pi}\left(s', \pi_{\tilde{\phi}}(s'); \tilde{\theta}\right)\right)^2$$

where $\tilde{\theta}$ is the target Q-function parameters and $\tilde{\phi}$ is the target policy function parameters. Both parameter values $\tilde{\theta}$ and $\tilde{\phi}$ are obtained from the last iteration. We call

$$target(s,a,s') = r(\mathrm{s},a) + \gamma Q^{\pi}\left(s', \pi_{\tilde{\phi}}(s'); \tilde{\theta}\right)$$

as the target value and $Q^{\pi}(s,a;\theta) - \ target(s,a,s')$ as *TD error*. Ideally, we want the error to decrease, meaning that our current policy's outputs are becoming more similar to the true Q values. Then, by differentiating the loss function with respect to the parameters θ, we have the gradient,

$$\nabla_{\theta}\ell_{\theta}(s,a,s') = \left(Q^{\pi}(s,a;\theta) - \mathrm{target}(s,a,s')\right)\nabla_{\theta}Q^{\pi}(s,a;\theta). \tag{7}$$

The policy learning step in DDPG will obtain a deterministic policy $\pi_{\phi}(s)$ which gives the action maximizes $Q(s,a;\theta)$. Because the action space is continuous, we assume the Q-function is differentiable with respect to action parameters. We can perform the gradient ascent with respect to policy parameters,

$$\max_{\phi} \mathrm{E}_{s\sim\mathcal{D}}[Q(s,\pi_{\phi}(s);\theta)] \tag{8}$$

where the expectation is estimated by using the training set, denoted by D, of tuple (s,a,s',r) from the EHR data. Then, we update the parameters of the Q-function and the policy function by using the gradient estimates in Eq. (7) and (8) and obtain new parameters θ and ϕ. At the end of each iteration, we update the target network, i.e., Q function $Q^{\pi}\left(s', \pi_{\tilde{\phi}}(s'); \tilde{\theta}\right)$ in $target(s,a,s')$ and target policy by

$$\tilde{\theta} \leftarrow \rho\tilde{\theta} + (1-\rho)\theta$$

$$\tilde{\phi} \leftarrow \rho\tilde{\phi} + (1-\rho)\phi$$

where ρ is a hyperparameter between 0 and 1.

The Q-network model, a.k.a. critic network in our paper uses a multi-layer feed-forward architecture which evaluates each state-action pair (s,a). Specifically, the model architecture contains a state input layer followed by a dense layer with 32 neurons and an action input layer; they are concatenated and then followed by a 16-dimensional dense layer; the output layer is 1 dimensional with a linear activation function. The policy model, a.k.a. actor network uses a two-later neural network with the state input followed by 32-dimensional intermediate layer and 1-dimensional action output layer. We also use batch normalization [30] after each dense layer to standardize the unit of low dimensional features. It is particularly useful in healthcare data as most biomarkers and vital signs have different physical unit and characteristics by nature and even statistics of the same type may vary a lot across multiple patients. Batch normalization can fix this issue by normalizing every dimension across samples in one minibatch.

We used the early stopping [31, 32] to prevent overfitting. There are two metrics used as early stopping criteria: mean squared TD error and consistency of recommendations between physician and RL. First, since the objective of DDPG is to minimize the mean squared TD error (7), it is natural to use (7) as a metric. Second, as we did not want RL-oxygen to be too much different from the standard of care, we used the consistency of recommendations as another metric, which is defined by the mean square deviations between RL's and physicians' recommended oxygen flow rates. In the study, we noticed that this second metric tends to converge later than the TD error. Thus, during training, we monitored both metrics and set the early stopping criterion to be that "mean squared deviation is not improved in last 500 iterations".

Our training scheme is as follows:

1. Split the dataset into 4 groups (one hospital per fold)
2. For each unique group:
    1) Take the group as a hold out or test data set;
    2) Take the remaining groups as a training data set;
    3) Fit a model on the training set and evaluate it on the test set;
    4) Retain the evaluation score;
    5) Repeat this process until every group serves as the test set.
3. Then take the average of the recorded scores as the performance metric for the model.

In reinforcement learning, learning an optimal policy from observational data is referred as to offline RL [13]. This approach uses a set of one-step transition tuples: $D = \{(s_i, a_i, r_i, s_i'): i = 1, \ldots, |D|\}$ to estimate the Q-function $Q^{\pi}(s, a'; \theta)$ and the oxygen flow policy $\pi(s)$. The learning algorithm follows [23] with 64 batch size and 0.002 learning rates for both critic and actor network.

Missing Data Imputation

Our dataset contains a set of historically observed health states, but not every possible health state, and the time series data such as lab tests, vital signs, and oxygen flow rate are sampled unevenly. In order to learn an optimal policy, RL requires a way to estimate values in any state, including those not in the original data. As such, we imputed data for such states based on the information from nearby measurements using a linear interpolation method.

References

1. Cox DR, (1972) Regression models and life‐tables. Journal of the Royal Statistical Society: Series B (Methodological) 34: 187-202
2. Zou H, Hastie T, (2005) Regularization and variable selection via the elastic net. Journal of the royal statistical society: series B (statistical methodology) 67: 301-320
3. LaValle SM, Branicky MS, Lindemann SR, (2004) On the relationship between classical grid search and probabilistic roadmaps. The International Journal of Robotics Research 23: 673-692
